# The efficacy of hyaluronic acid fragments with amino acid in combating facial skin aging: an ultrasound and histological study

**DOI:** 10.1007/s40477-024-00925-5

**Published:** 2024-06-24

**Authors:** Antonio Scarano, E. Qorri, A. Sbarbati, S. A. Gehrke, Alessio Frisone, D. Amuso, Sergio Rexhep Tari

**Affiliations:** 1 Department of Medical, Oral and Biotechnological Sciences, University of Chieti-Pescara, Strada Marcello Mucci 38/B, 66100 Chieti, Italy; 2 Department of Dentistry, Faculty of Medical Sciences, Albanian University, 1001 Tirana, Albania; 3 Department of Neurosciences, Biomedicine and Movement Sciences, Anatomy and Histology Section, School of Medicine, University of Verona, Verona, Italy; 4 Department of Research, Bioface/PgO/UCAM, Montevideo, Uruguay

**Keywords:** Hyaluronic acid, Hyaluronic acid fragments, Amino acid, Fibroblast, Skin aging, Ultrasound

## Abstract

**Background:**

Various techniques have been employed in aesthetic medicine to combat skin aging, in particular that of the facial region. Hyaluronic acid is utilized to enhance moisture levels and extracellular matrix molecules. This study aims to histologically assess the effects of low molecular weight hyaluronic acid fragments combined with amino acids (HAAM) on facial skin rejuvenation through intradermal microinjections.

**Methods:**

A total of twenty women, with an average age of 45 and ranging from 35 to 64 years old, participated in the study, including 8 in menopause and 12 in the childbearing age group. Mesotherapy was used to administer HAAM to the patients. Prior to and three months after the treatment, each patient underwent small circular punch biopsies. Ultrasound examinations were conducted using B-mode, capturing 2D images in longitudinal or transverse orientations with frequencies ranging from 5 to 13 Mega-hertz (MY LAB X8, ESAOTE, Genova, Italy). A total of 60 ultrasound examinations were taken, with 30 collected before treatment and 30 after treatment.

**Results:**

The histological analysis demonstrates an increase in fibroblast activity resulting in the production of Type III reticular collagen, as well as an increased number of blood vessels and epidermal thickness. However, the analysis of ultrasound data before and after treatment showed no statistical difference in skin thickness in malar area, chin and mandibular angle.

**Conclusions:**

Histological assessments indicate that subcutaneous infiltration of HAAM has a substantial impact on the dermis of facial skin.

## Introduction

Human skin, aside from its vital protective function, plays a delicate role in facilitating metabolic and informational exchanges between the body and its surrounding environment. Regrettably, due to various external factors (e.g., ultraviolet radiation) and internal stressors (e.g., endocrine-metabolic disorders), compounded by the relentless passage of time, it can experience a series of functional alterations and structural issues that may compromise its integrity to varying degrees [1]. These can be seen in skin discoloration, dehydration, reduced elasticity, and microvascular changes, among others, particularly noticeable when affecting critical areas like the face and décolleté [2, 3]. To address and prevent such skin imperfections, including but not limited to wrinkles, the concept of “cutaneous biostimulation” has been proposed in the realm of cosmetic treatments. This approach relies on injections aimed at regenerating the normal structure and functions, of the skin, primarily by targeting the fibroblasts that make up its framework and the surrounding extracellular matrix [4].

However, it is essential to recognize that the fibroblast-myofibroblast-fibrocyte complex is just one piece of a much larger puzzle, encompassing other components, both cellular (such as blood and lymphatic vessel endothelium, monocyte-macrophages, lymphocytes, nerve ganglia, etc.) and non-cellular (like the extracellular matrix and others). Their anabolic processes need to be continually balanced by catabolic processes and cellular recycling, all under the control of signaling molecules (ranging from free radicals to cytokines and growth factors) [5, 6]. In other words, given the intricate molecular-level structure and function of the skin, it is evident that stimulating fibroblasts alone may prove insufficient for preventing or treating imperfections without an "integrated" approach.

In the field of aesthetic medicine, a noteworthy observation is that hyaluronic acid fragments containing 20 to 30 monomers can activate fibroblasts by binding to CD44 receptors, thereby promoting the production of reticular collagen type III [7, 8]. Naturally, this phenomenon is contingent on the availability of precursors, enzymes, and cofactors involved in the biosynthesis of this structural protein, as elaborated upon later in this text [9–11]. Hyaluronic acid is principal component of the extracellular matrix, that plays a important role in preserving tissue structure and regulating cell signaling pathways. HA offer protection against oxidative stress, preserve skin elasticity, improve wound healing and resistance to cancer and arthritis, largely attributable to CD44 signaling and other intricate mechanisms [12, 13]. Following previous clinicals trials, hyaluronic acid mixed with amino acids was investigated to counter skin ageing [11, 14]. Ultrasonography (US) has used in diverse applications in research aspects, clinical and surgical aspect. Alexander and Miller in 1979 used a one-dimensional 15 MHz device to measure skin thickness [15]. Today higher frequency machines have been developed, with the goal of improving resolution and seeing surface features even at shorter wavelengths [16]. US have been increasingly adopted in various areas of medicine due to painlessness and cost-effectiveness. This study explores the role of hyaluronic acid alongside various organic and inorganic substances in preventing and treating common skin imperfections. The objective of this study is to conduct a histological and ultrasound assessment of the impact of low molecular weight hyaluronic acid fragments and amino acids (HAAM) on the rejuvenation of facial skin through intradermal microinjections.

## Materials and methods

A total of twenty women, with an average age of 45 (ranging from 35 to 64), were included in this study, comprising eight in menopause and twelve in the childbearing age group. The study took place at the Department of Medical Sciences of the University of Tirana, Albania, registered with Nr.321 Prot. date 24.05.2022. It adhered rigorously to ethical principles, including compliance with the World Medical Association Declaration of Helsinki (https://www.wma.net/wp-content/uploads/2018/07/DoH-Oct2008.pdf) and the additional requirements stipulated by Albania law. The clinical investigation plan (CIP) for this study was developed in accordance with the requirements of the International Standard ISO 14155. All patients provided informed consent for the procedure, although they were unaware of the specific condition being treated. Common issues observed among all patients included generalized rhytidosis, inadequate skin hydration, and diminished sebum production under dry, hypoxic conditions. Exclusion criteria comprised changes in diet, pregnancy, a history of heavy smoking (20 cigarettes per day), a history of allergic and/or irritant contact hand dermatitis, systemic diseases, and psychiatric illnesses.

**Fig. 1:**
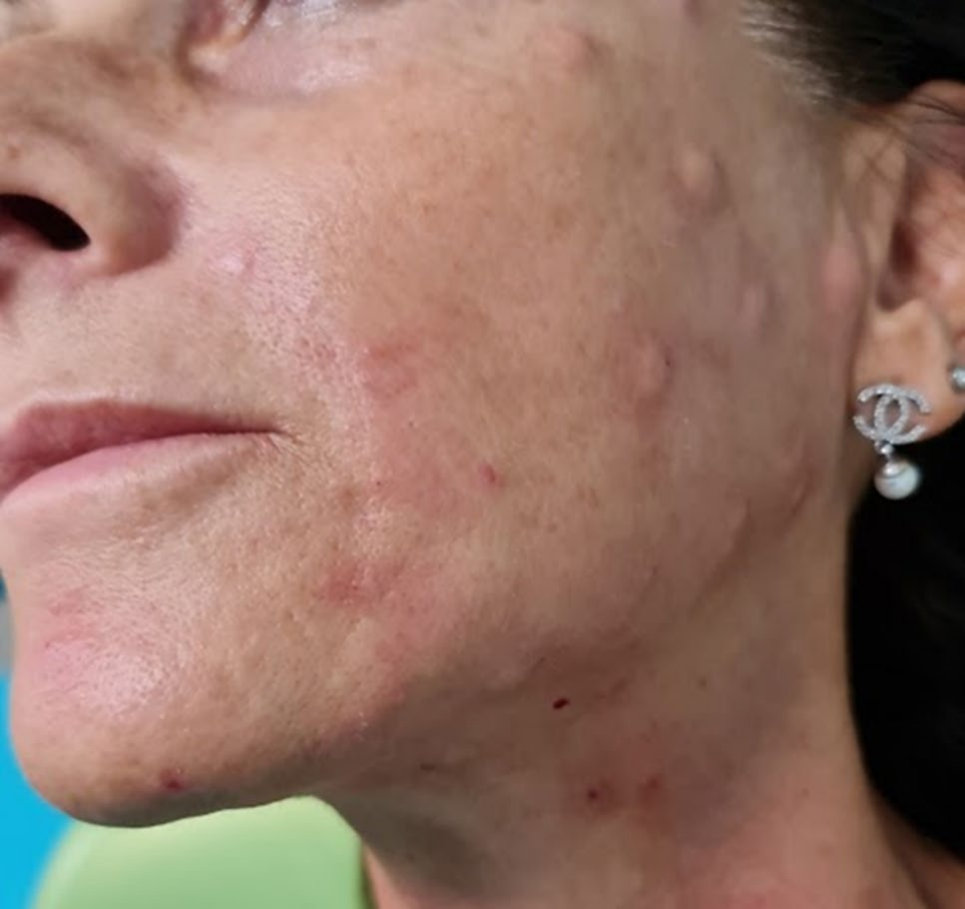
Clinical aspect immedialtely after iniection of HAAM

Following the collection of anamnestic data and a physical examination, patients underwent pH measurement, sebometry, hydrometry, photography, and echography assessments in regions including the glabella, canthal, malar-cheek, chin, and neck. SoftPlus (Callegari, Parma, Italy) was utilized to assess skin hydration, sebum production, and pH levels, with hydration measured in g/m^2^/h and sebum production in µg/cm^2^/min. These clinical results were published in a previous study [11]. The study employed a common base of low molecular weight hyaluronic acid fragments, to which a buffered aqueous solution was added. The solution contained Sodium Hyaluronate along with l-lysine, l-proline, l-alanine, glycine, l-serine, l-cysteine, l-leucine, l-valine, and l-isoleucine buffer in a solution with Sodium bicarbonate (SKIN-B^®^ Italfarmacia, Rome, Italy). Patients received treatment with HAAM products using mesotherapy techniques, following an 8-week protocol. The solution was injected into the deep dermal layer, with a minimum of 0.2/0.3 mL administered at hypotonic points. A 2.5 mL syringe was used for precise dosing, along with a meso needle 30G × 6 mm. Patients underwent one session every 15 days, totaling four sessions (Fig. 1).

### Ultrasound examinations

Before and three months after the therapeutic procedure, each patient with Ultrasound. Ultrasound examinations were conducted using B-mode, capturing 2D images in longitudinal or transverse orientations with frequencies ranging from 5 to 13 MHz (MY LAB X8, ESAOTE, Genova, Italy). These assessments allowed visualization of the epidermis, dermis, and subcutaneous structures. Morphological characteristics, ephitelium thickness and dermal thickness were evaluated in regions including the malar-cheek, chin, and mandibular angle. Dermal thickness and epithelium thickness were measured by drawing a straight line perpendicular to the epithelium and the subcutaneous tissue using Viewer software. A total of 60 ultrasound examinations were taken, with 30 collected before treatment and 30 after treatment.

### Histological evaluation

**Fig. 2:**
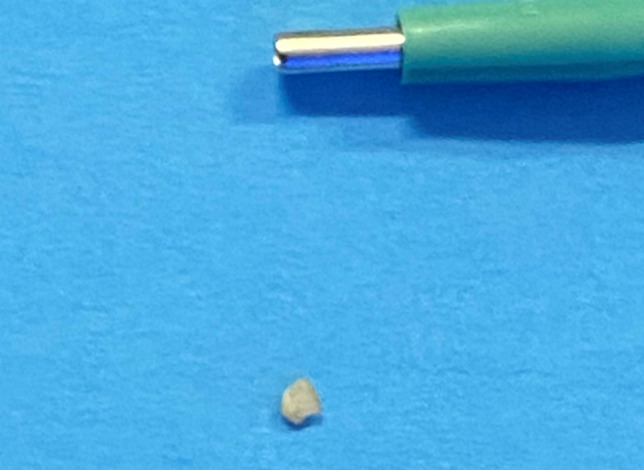
Small biopsies were taken before and three months after the therapeutic procedure

Before and three months after the therapeutic procedure, each patient underwent small 2-mm diameter circular punch biopsies (KAI Industries, Oyana Japan) (Fig. 2). The biopsy was retrieved only in angular mandibular area. Before treatment the specimens were retrieved in left mandibular angle area, while before treatment were retrieved in right mandibular angle area. This to avoid to analysis the same area and relative artefacts.

A total of 40 biopsies were taken, with 20 collected before treatment and 20 after treatment.

These specimens were stained with Hematoxylin and Eosin, Masson’s trichrome, and Van Gieson. Four fields, each measuring 2000 μm in diameter and 4000 μm in length, were evaluated for each sample [17].

A specific histological evaluation was conducted using HAAM on a sample of subjects with skin imperfections. The immunohistochemical reaction to Epidermal Growth Factor (EGF) was quantified, excluding the stratum corneum when measuring the epidermis. The immunohistochemical reaction to Vascula Endotelial Growth Factor (VEGF) was quantified, excluding the stratum corneum when measuring the epidermis.

Histological evaluations of the biopsy slides were conducted by two pathologists from different medical universities in Verona and Modena Reggio Emilia to minimize the potential for interpretation bias.

The biopsies were carried out to document:

- Epithelial thickness
- Dermal thickness
- Number of blood vessels
- Number of cells positive for epidermal growth factor (EGF)

### Statistical analysis

A power analysis was performed using clinical software to determine the required number of samples for statistical significance in quantitative analyses of cell numbers, including epithelial thickness, positive Epidermal Growth Factor (EGF) cells, and vessel numbers. A calculation model was employed for dichotomous variables (yes/no effect) to discern the reasons, with a 90% incidence effect for the Test group and 10% for the control group, at an alpha level of 0.05 and power level of 80%. The optimal number of samples for analysis was determined to be 10 patients per group.

Numerical results were presented as means with standard deviations.

The study data were collected and evaluated by the Graphpad 6 (Prism, San Diego-CA USA) statistical software package. The Kolmogorov–Smirnov test was performed to evaluate the normal distribution of the study data were normally distributed and the t-Student test was used to calculate the statistical significance among the variables investigated.

## Results

### Ultrasound examinations

**Fig. 3:**
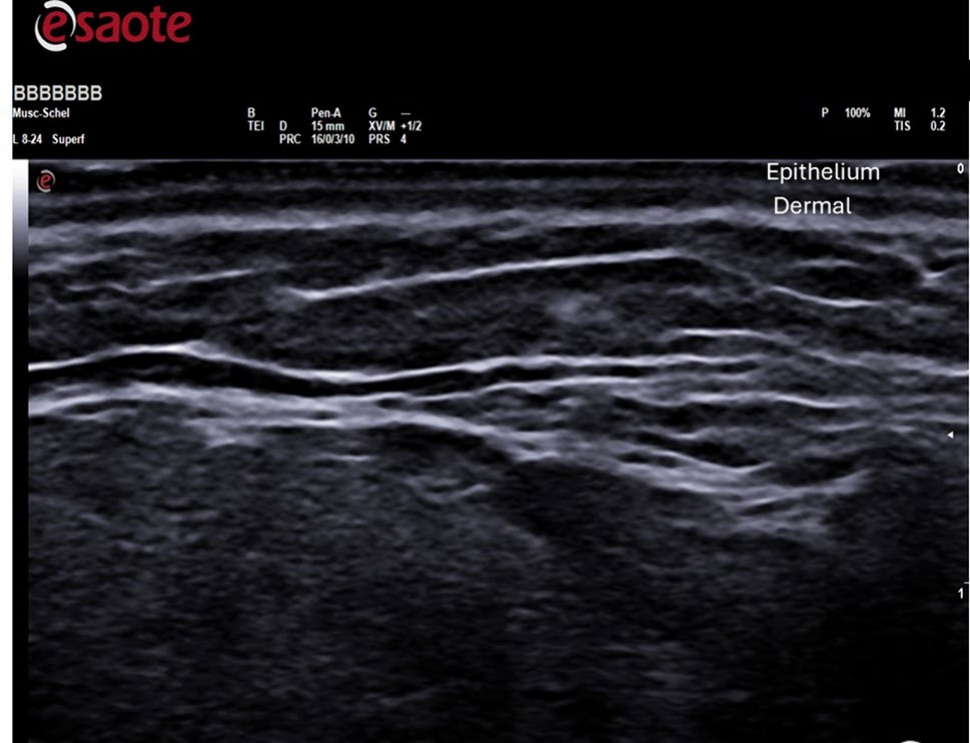
Ultrasound evalutation: the graphs show dermal thickness, epithelium thickness before and after treatment—Ultrasound images before treatment

**Fig. 4:**
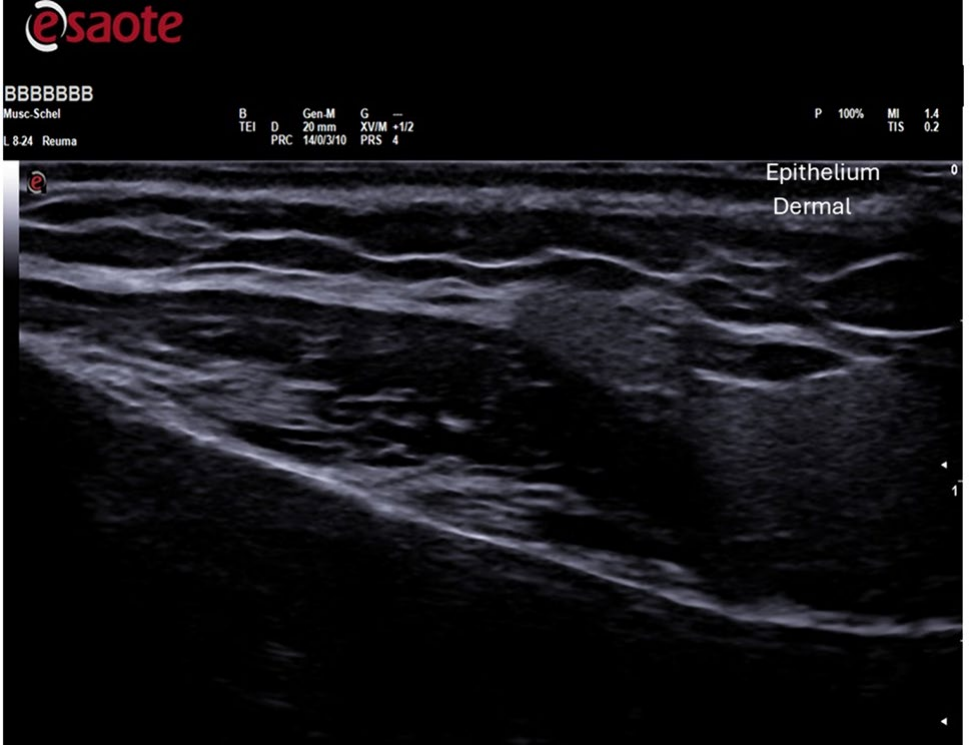
Ultrasound evalutation: the graphs show dermal thickness, epithelium thickness before and after treatment—Ultrasound images after treatment

Skin, epidermal, and dermal thicknesses were not significantly correlated with treatment with HAAM. Analysis of data before and after treatment showed no statistical difference in skin thickness in malar area, chin and mandibular angle. In the images acquired with the ultrasound scanner, the three upper layers of the skin were clearly distinguished: epidermis, dermis and subcutaneous fat layer. The data are summarized in the Table 1 and Figs. 3 and 4

**Table 1:** Summary of the effectiveness before and after the biomodulation treatment (mean, standard deviation)

Groups	Dermal thickness (mm)		Epidermis thickness (µm)	
	Before	After	Before	After
MC (malar-cheek)	1.11 ± 0.593	1.219 ± 0.640	0.127 ± 0.080	0.201 ± 0.584
*p* value	*p* = 0.58		*p* = 0.52	
GC (Chin)	1.263 ± 0.375	1.327 ± 0.403	0.149 ± 0.080	0.289 ± 0.289
*p* value	*p* = 0.6650		*p* = 0.0344	
MA (mandibular angle)	1.243 ± 0.535	1.313 ± 0.656	0.102 ± 0.072	0.232 ± 0.619
*p* value	*p* = 0.6344		*p* = 0.3657

### Histological assessment

#### Before treatment

**Fig. 5:**
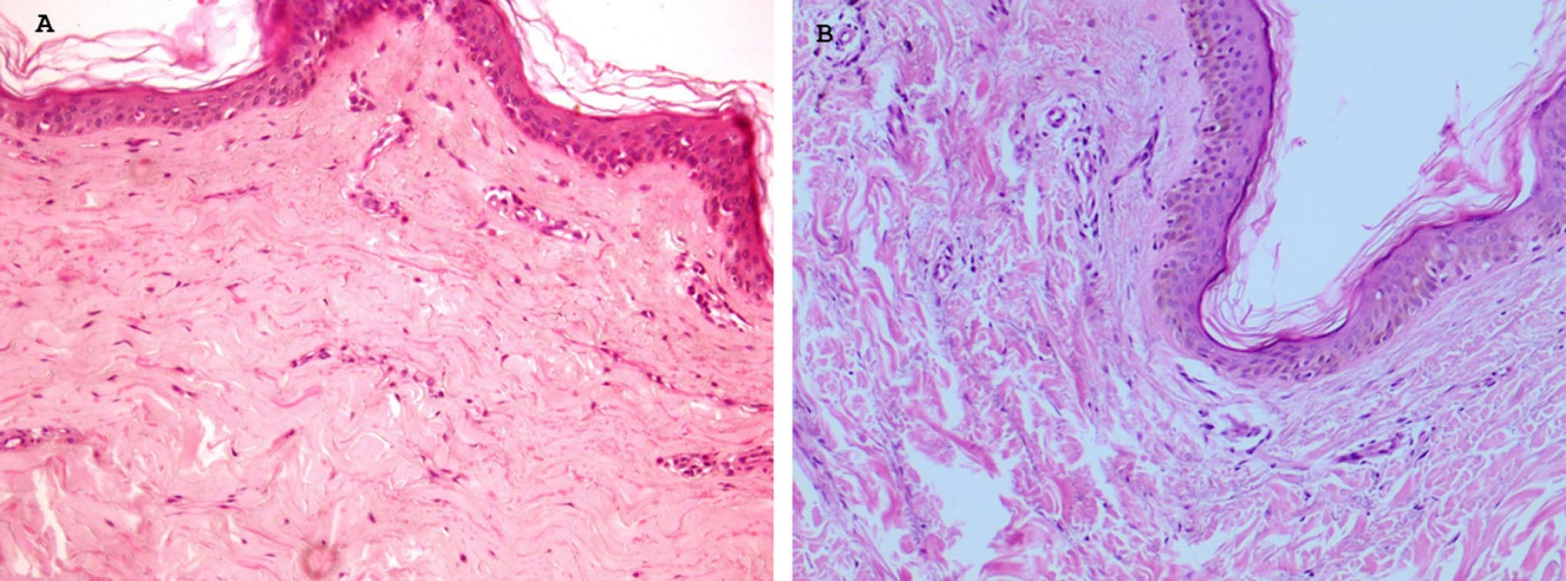
**A** The epidermis exhibited a well-structured. **B** The appearance of the epidermis is characterised by increased thickness. Hematoxylin and eosin 30×

The biopsies displayed a loss of collagen bundles in the reticular layer, along with evident microcirculation alterations. A limited number of fibroblasts and reduced epidermal appearance consistent with certain microcirculation components were observed (Fig. 5B). No pathological inflammatory cells observed in the epithelium or epidermis (Fig. 5A and B). The histomorphometric results are presented in the accompanying table.

#### After treatment

**Fig. 6:**
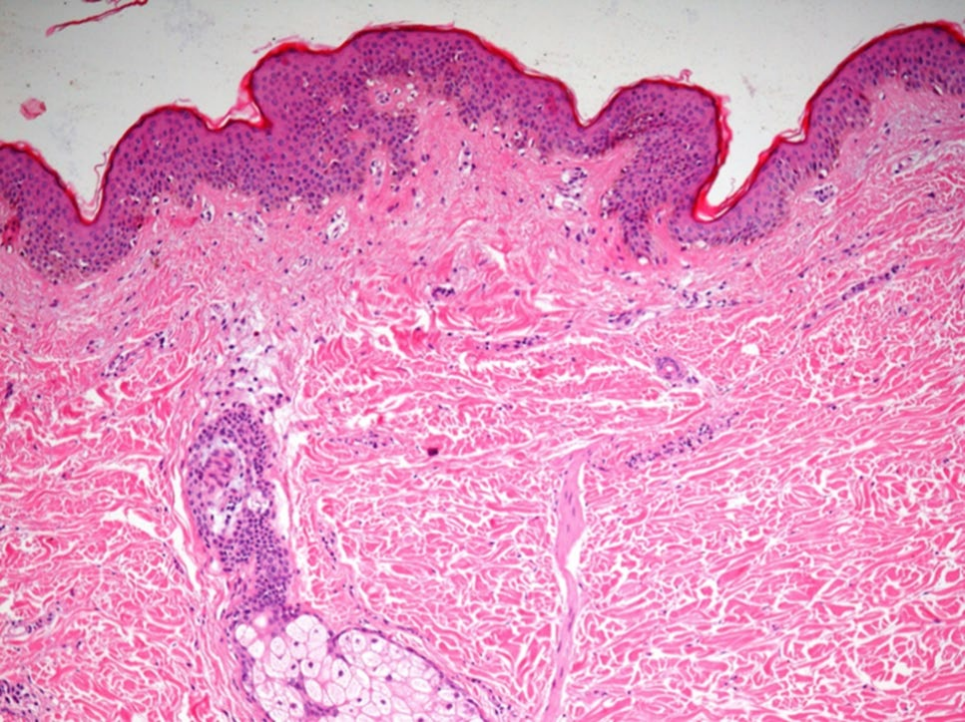
After treatment, there is an increase in vessels, fibroblasts and thickness epithelium.  Hematoxylin and eosin 20×

**Fig. 7:**
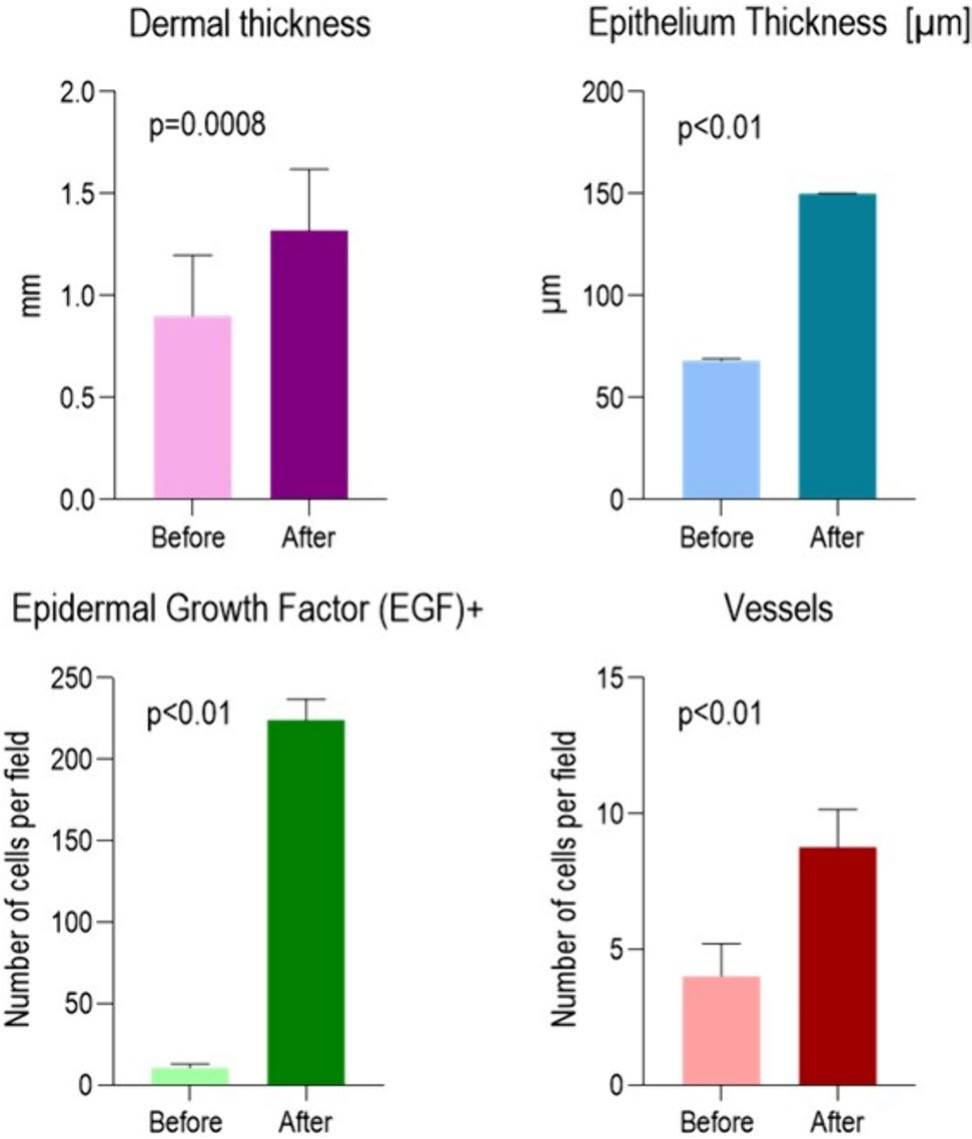
Histological evaluations: the graphs show dermal thickness, epithelium thickness, epidermal growth factor and vessel before and after treatment

**Fig. 8:**
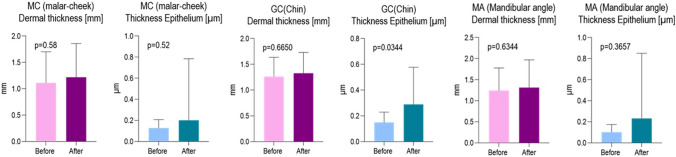
Ultrasound evalutation: the graphs show dermal thickness, epithelium thickness before and after treatment

These results clearly demonstrate that injectable HAAM treatments are capable of stimulating fibroblast activity, leading to the production of Type III reticular collagen. The epidermis exhibited a well-structured appearance with increased thickness (see Fig. 6). Notably, there was evident replicative activity of the epidermis, confirmed by positive immuno-histochemical reactions to Epidermal Growth Factor (EGF). Fibroblasts displayed immunohistochemical positivity in the nuclei of the germinative layer of the epidermis (basal layer) and in certain dermal cells. Collagen fibers appeared visibly reorganized, and microcirculation was evident. Collagen fiber content in the epidermis increased noticeably, as did epidermal thickness, all without the presence of pathological inflammatory cells. Detailed histomorphometric results are provided in the Table 2 and Figs. 7 And 8.

**Table 2:** Summary of the effectiveness before and after the biomodulation treatment (mean, standard deviation)

Groups	Dermal thickness [mm]		Epithelium thickness [µm]		Epidermal Growth factor (EGF)^+^		Vessels	
	Before	After	before	after	before	after	Before	After
Average	0.9	1.32	68.21	150.01	10.5	224.0	4.0	8.75
SD	± 0.3	± 0.3	± 1.1	± 0.3	± 2.22	± 12.77	± 1.2	± 1.4
*p* value	*p* = 0.0008		*p* < 0.01		*p* < 0.01		*p* < 0.01

## Discussion

The results of the current study's histological findings are undeniably positive, while ultrasound investigations showed no significant differences. Skin, epidermal and dermal thicknesses assessed by the US did not vary appreciably in thickness. According to histological evidence, the stratum corneum (SC) seems to keep its typical thickness, although old skin has a thinner epidermis than younger skin, which is explained by the retraction of rete ridges [18]. These data are confirmed with US studies that showed similar results for SC thickness. However, identification of the precise alterations with US that occur during the aging process is still difficult, despite much research on epidermal, dermal, and total skin thickness [19].

Indeed, the objective has been met, specifically, to demonstrate that the utilization of medical devices containing hyaluronic acid fragments ranging from 20 to 38 monomers, in conjunction with amino acids via dermal injection techniques, leads to noticeable enhancements in the dermal and epidermal structure of facial skin. The administration of HAAM has a discernible impact on epidermal hydration, signifying the availability of increased moisture for standard physiological processes.

The histological assessment revealed a heightened activity of fibroblasts, resulting in the production of Type III reticular collagen. Moreover, there was a notable increase in the number of blood vessels, and the epidermis exhibited greater thickness, accompanied by an augmentation in the basal stratum cell count. Collagen fibers became visibly reorganized, and microcirculation was evident. Importantly, there was a significant increase in collagen fiber content within the epidermis, and the thickness of the epidermal layer saw growth without any pathological presence of inflammatory cells.

Hyaluronate (HA) has been widely employed in aesthetic medicine for procedures related to soft tissue augmentation and skin rejuvenation [20]. HA is naturally synthesized on the cell membranes of various cell types, including fibroblasts and keratinocytes. It plays a vital role in maintaining hydration levels within medium connective tissues, facilitating cellular movement during inflammation and supporting wound healing [21]. Decreased HA levels are associated with skin aging [22].

In this study, hyaluronic acid fragments ranging from 20 to 38 monomers were chosen, combined with amino acids, due to their significant role in dermal biomodulation [23–27]. Hyaluronic acid constitutes the most prevalent glycosaminoglycan in the extracellular matrix and achieves its highest concentration in highly hydrated tissues such as vitreous fluid and the umbilical cord [28]. Enzymatic hyaluronate degradation into fragments by hyaluronidases stimulates processes like angiogenesis, tissue remodeling, and cell turnover [29, 30].

The biological effects of hyaluronate fragments (HF) are influenced by their length. Longer HF, exposed to more receptor sites, demonstrate higher receptor avidity, resulting in enhanced binding [31, 32]. An HF containing 20 monomers, comprising two hexamers spaced by an octamer (likely in a favorable conformation, such as helicoidal), each binding to a CD44 receptor, achieves optimal efficacy [33]. HF equal to or exceeding 30 units do not provide significantly improved binding [34, 35]. Shorter HF exhibit variable effects, influencing the modulation of inflammatory processes and immune responses by activating various receptors, including RHAMM, Toll-like receptors (TLRs), Hyaluronan Receptor for Endocytosis (HARE), and Lymphatic Vessel Endothelial Receptor 1 (LYVE-1) [11, 36–53].

In the present study, amino acids were utilized to stimulate protein synthesis. It is plausible that HAAM induces a pro-inflammatory state during an initial phase, which serves a functional purpose in the healing process. It is important to note that at the highly acidic pH of the device, amino acids do not undergo chemical modification but rather experience changes in their electrolytic properties. This includes a reordering of hydrogen ions, resulting in mainly protonated forms that carry a positive charge. The loss of polarity potentially facilitates their uptake by fibroblasts [54]. The gradual diffusion of hydrogen ions from the acidic formulation subsequently leads to the gradual hydrolysis of the hyaluronic acid fragments. Consequently, following a transient pro-inflammatory phase, dermal reactivity is physiologically modulated in an anti-inflammatory direction, effectively contributing to tissue repair.

From a microcirculation perspective, an increase in both acute and chronic perfusion capacity within the capillary bed becomes evident. This enhancement may be linked to structural alterations in perivascular and preadipocyte structures [55], likely associated with functional and biochemical modifications, as demonstrated in histological tests. However, ultrasound investigations showed no significant differences. Some limitation of the present investigation is that relatively low ultrasound frequencies were used. We do not know whether using higher frequencies would have resulted in different ultrasound findings. Due to its noninvasive, inexpensive, and safe nature, ultrasound is a technology that has promising future prospects in antiaging and aesthetic medicine. Another limitation of this work are those related to the low number of patients, which should be the subsequent extensions of this work. Based on these observations, it is theorized that low molecular weight hyaluronic acid fragments, through their stimulation of the inflammatory cascade and the immune system, along with the reduction in the expression of matrix metalloproteinases MMP-1 and MPP-3, and their ability to stimulate angiogenesis and control oxidative stress, thus facilitating tissue regeneration processes during the acute phase. This confers upon hyaluronic acid the role of a genuine physiological modulator. This study shows that low HA fragments and amino acids contribute to positive histological changes in dermis of the face.

## Conclusions

In conclusion the histological results shows that the administration of HAAM via injection techniques stimulates fibroblasts and leads to aesthetic improvements in the appearance of the faces of the patients treated. However, ultrasound examinations conducted using B-mode, capturing 2D images in longitudinal or transverse orientations with frequencies ranging from 5 to 13 MHz do not show any changes.

## Data Availability

All experimental data to support the findings of this study are available contacting the corresponding author upon request.
